# Human skeletal muscle metabolic responses to 6 days of high‐fat overfeeding are associated with dietary n‐3PUFA content and muscle oxidative capacity

**DOI:** 10.14814/phy2.14529

**Published:** 2020-08-26

**Authors:** Sophie L. Wardle, Lindsay S. Macnaughton, Chris McGlory, Oliver C. Witard, James R. Dick, Philip D. Whitfield, Arny A. Ferrando, Robert R Wolfe, Il‐Young Kim, D. Lee Hamilton, Colin N. Moran, Kevin D. Tipton, Stuart D. R. Galloway

**Affiliations:** ^1^ Physiology, Exercise and Nutrition Research Group University of Stirling Stirling UK; ^2^ Army Health and Performance Research, Army Headquarters Andover UK; ^3^ Sportscotland Institute of Sport Stirling UK; ^4^ Queens University Kingston Ontario Canada; ^5^ Centre for Human and Applied Physiological Sciences King’s College London London UK; ^6^ Nutrition Group, Institute of Aquaculture University of Stirling Stirling UK; ^7^ Lipidomics Research Facility, Division of Biomedical Sciences University of the Highlands and Islands Inverness UK; ^8^ Department of Geriatrics, Center for Translational Research in Aging and Longevity Donald W. Reynolds Institute on Aging Little Rock AR USA; ^9^ Institute for Physical Activity and Nutrition (IPAN), School of Exercise and Nutrition Sciences Deakin University Geelong Australia; ^10^ Department of Sport and Exercise Sciences, Faculty of Social Sciences and Health Durham University Durham UK

**Keywords:** exercise, fish oil, insulin resistance, omega‐3, overfeeding, type 2 diabetes

## Abstract

Understanding human physiological responses to high‐fat energy excess (HFEE) may help combat the development of metabolic disease. We aimed to investigate the impact of manipulating the n‐3PUFA content of HFEE diets on whole‐body and skeletal muscle markers of insulin sensitivity. Twenty healthy males were overfed (150% energy, 60% fat, 25% carbohydrate, 15% protein) for 6 d. One group (*n* = 10) received 10% of fat intake as n‐3PUFA rich fish oil (HF‐FO), and the other group consumed a mix of fats (HF‐C). Oral glucose tolerance tests with stable isotope tracer infusions were conducted before, and following, HFEE, with muscle biopsies obtained in basal and insulin‐stimulated states for measurement of membrane phospholipids, ceramides, mitochondrial enzyme activities, and PKB and AMPKα2 activity. Insulin sensitivity and glucose disposal did not change following HFEE, irrespective of group. Skeletal muscle ceramide content increased following HFEE (8.5 ± 1.2 to 12.1 ± 1.7 nmol/mg, *p* = .03), irrespective of group. No change in mitochondrial enzyme activity was observed following HFEE, but citrate synthase activity was inversely associated with the increase in the ceramide content (r=−0.52, *p* = .048). A time by group interaction was observed for PKB activity (*p* = .003), with increased activity following HFEE in HF‐C (4.5 ± 13.0mU/mg) and decreased activity in HF‐FO (−10.1 ± 20.7 mU/mg) following HFEE. Basal AMPKα2 activity increased in HF‐FO (4.1 ± 0.6 to 5.3 ± 0.7mU/mg, *p* = .049), but did not change in HF‐C (4.6 ± 0.7 to 3.8 ± 0.9mU/mg) following HFEE. We conclude that early skeletal muscle signaling responses to HFEE appear to be modified by dietary n‐3PUFA content, but the potential impact on future development of metabolic disease needs exploring.

## Graphical abstract

High‐fat high‐energy diets contribute to development of insulin resistance and type 2 diabetes, and n‐3PUFA have been proposed to improve insulin sensitivity. We examined the potential for dietary n‐3PUFA to mitigate negative metabolic consequences of short‐term high‐fat overfeeding in healthy adult males. We report that short‐term high‐fat energy excess over 6 d did not lead to whole body changes in glucose handling, but changes at the cellular level did occur, including an increase in muscle ceramide species. We show that substituting 10% of total fats for n‐3PUFA during a short term high‐fat high‐energy diet appears to lead to early differential modifications in cellular signalling responses linked to metabolic health outcomes, but these findings require replication and further study.

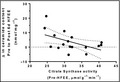

## 1 INTRODUCTION

Dietary energy and fat overconsumption leading to body mass gain is associated with the development of obesity and insulin resistance (Chan et al., 1994; Colditz et al., 1995). High‐fat, high‐energy diets have become increasingly common, particularly in Western countries, and are thought to contribute to insulin resistance and type 2 diabetes (T2D) (Van Dam et al., 2002). Investigation of the early physiological responses of humans to dietary fat and energy excess is important to improve our understanding of the chronic (mal)adaptations that lead to obesity, insulin resistance, and T2D, and to inform the development of targeted preventative strategies to address these early responses.

Short‐term consumption of a high‐fat energy excess (HFEE) diet is a model that has been widely used to investigate mechanisms leading to diet‐induced insulin resistance. Experimental studies that increased dietary intake of saturated fats (SFA) demonstrate reductions in whole‐body insulin sensitivity (Bachmann et al., 2001; Hulston et al., 2015; Parry et al., 2017; Parry et al., 2019). However, studies using equal proportions of the three main classes of fats (SFA, monounsaturated (MUFA), and polyunsaturated (PUFA)) have failed to demonstrate an influence of overfeeding on whole‐body measures of insulin sensitivity (Adochio et al., 2009; Brøns et al., 2009; Cornier et al., 2006). While a diet rich in SFA is typically related to the development of obesity and T2D (Van Dam et al., 2002), consuming long chain n‐3 polyunsaturated fatty acids (n‐3PUFAs) appears to have clinical benefit in reducing the development of insulin resistance (Fedor & Kelley, 2009). Hence, there is clear rationale that the fat composition of HFEE diets could modulate the metabolic consequences of overfeeding.

At the cellular level, an accumulation of lipid moieties including ceramides, diacylglycerols, and long chain fatty acyl‐CoAs in insulin‐receptive skeletal muscle tissue characterizes insulin resistance (Holland et al., 2007; Zierath, 2007). The accumulation of ceramides in skeletal muscle of obese, insulin‐resistant individuals is coupled with a downregulation of insulin signaling, including decreased phosphorylation of PKB^Ser473^ and PKB^Thr308^ (Adams et al., 2004), and impaired mitochondrial function (Holloway et al., 2009), possibly driven by reduced AMPK activity (O'Neill et al., 2012; Steinberg et al., 2006). The pharmacological activation of AMPK has been shown to increase fatty acid oxidation (Dzamko & Steinberg, 2009; O’Neill et al., 2012), whereas reduced AMPK activity is associated with decreased fatty acid oxidation within skeletal muscle (O’Neill et al., 2012; Steinberg et al., 2006). Interestingly, mitochondrial oxidative capacity is increased in response to HFEE in animal models (Hancock et al., 2008; Jain et al., 2014; Turner et al., 2007), but this mitochondrial upregulation has not been demonstrated in humans (Samocha‐Bonet et al., 2012; Toledo et al., 2018). Thus, developing a better understanding of the relationships between HFEE, skeletal muscle oxidative capacity, and lipid‐induced changes in ceramides and insulin signaling will provide novel insights into metabolic handling of HFEE in humans.

Dietary fish oil‐derived n‐3PUFAs may be protective against the responses typically observed with the development of insulin resistance (Albert et al., 2014; Souza et al., 2020), but few studies have explored their role alongside short‐term high‐fat overfeeding. Dietary fish oil has a triglyceride lowering effect (Harris, 1996, 1997), even with high‐fat diets (Turvey et al., 2005), and the n‐3PUFA composition of skeletal muscle membranes positively correlates with insulin sensitivity (Borkman et al., 1993). Eicosapentanoic acid (EPA) and docosahexanoic acid (DHA) fish oil supplement strategies in previous studies have varied from 4g/d over 8 weeks (Smith et al., 2011) to 5g/d over 4 weeks (McGlory et al., 2014), and even 50g/d over only 3 days (Turvey et al., 2005). All of these studies have observed increases in muscle n‐3PUFA phospholipid fractions and/or skeletal muscle metabolic effects. Thus, evaluating the influence of n‐3PUFA intake on early adaptations to HFEE using a high‐dose, short‐term fish oil ingestion approach will inform our understanding of the influence of dietary fat composition, during high‐fat overfeeding, on the regulation of glucose and fat metabolism.

The primary aim of this study was to evaluate short‐term changes in whole‐body glucose handling and insulin sensitivity following HFEE with or without high‐dose fish oil supplementation. The secondary aim was to investigate the muscle lipid composition and activity of intracellular signaling proteins known to regulate metabolism following short‐term HFEE. Both of these aims were designed to understand the impact of manipulating dietary n‐3PUFA content on the metabolic response to short‐term HFEE. We hypothesized that: 1) HFEE for 6 d would reduce whole‐body insulin sensitivity/ glucose disposal, through alterations in skeletal muscle lipid composition that would negatively affect the activity of intracellular proteins involved in insulin signaling, and; 2) substitution of 10% of total fats with dietary n‐3PUFAs obtained from fish oil would offset these negative responses.

## 2 METHODS

### 2.1 Participants and ethical approval

Twenty healthy, young, moderately active men with no family history of T2D were recruited from the University of Stirling and surrounding area (Table 1). Following health screening and dietary analysis, participants were excluded from the study if their fasting blood glucose concentration was > 6.1 mmol/L, they habitually consumed a diet high in n‐3PUFA sources, consumed n‐3PUFA supplements, exercised for more than 150 min per week, or were actively trying to gain or lose body mass. Ethical approval was obtained from the NHS East of Scotland Research Ethics Service (REC 2) and the study was conducted in accordance with the Declaration of Helsinki. Participants provided written informed consent for their participation once the study purpose and procedures had been explained in lay terms.

**Table 1:** Participant characteristics on entry into the study for high‐fat control (HF‐C, *n* = 10) and high‐fat fish oil (HF‐FO, *n* = 10) groups

	HF‐C	HF‐FO
Age (y)	21.8 ± 3.4	22.9 ± 3.7
Body mass (kg)	72.4 ± 7.7	70.1 ± 7.9
BMI (kg·m^−2^)	21.8 ± 2.0	21.3 ± 2.0
Physical activity (h/wk)	1.1 ± 0.4	1.2 ± 0.4
Fasting insulin (µIU/ml)	12.6 ± 5.1	11.1 ± 2.9
Fasting glucose (mM)	5.0 ± 0.3	4.9 ± 0.4

Note

Values are means ± *SD*. No differences were observed in any parameter between groups.

### 2.2 Study design

**Figure 1:**
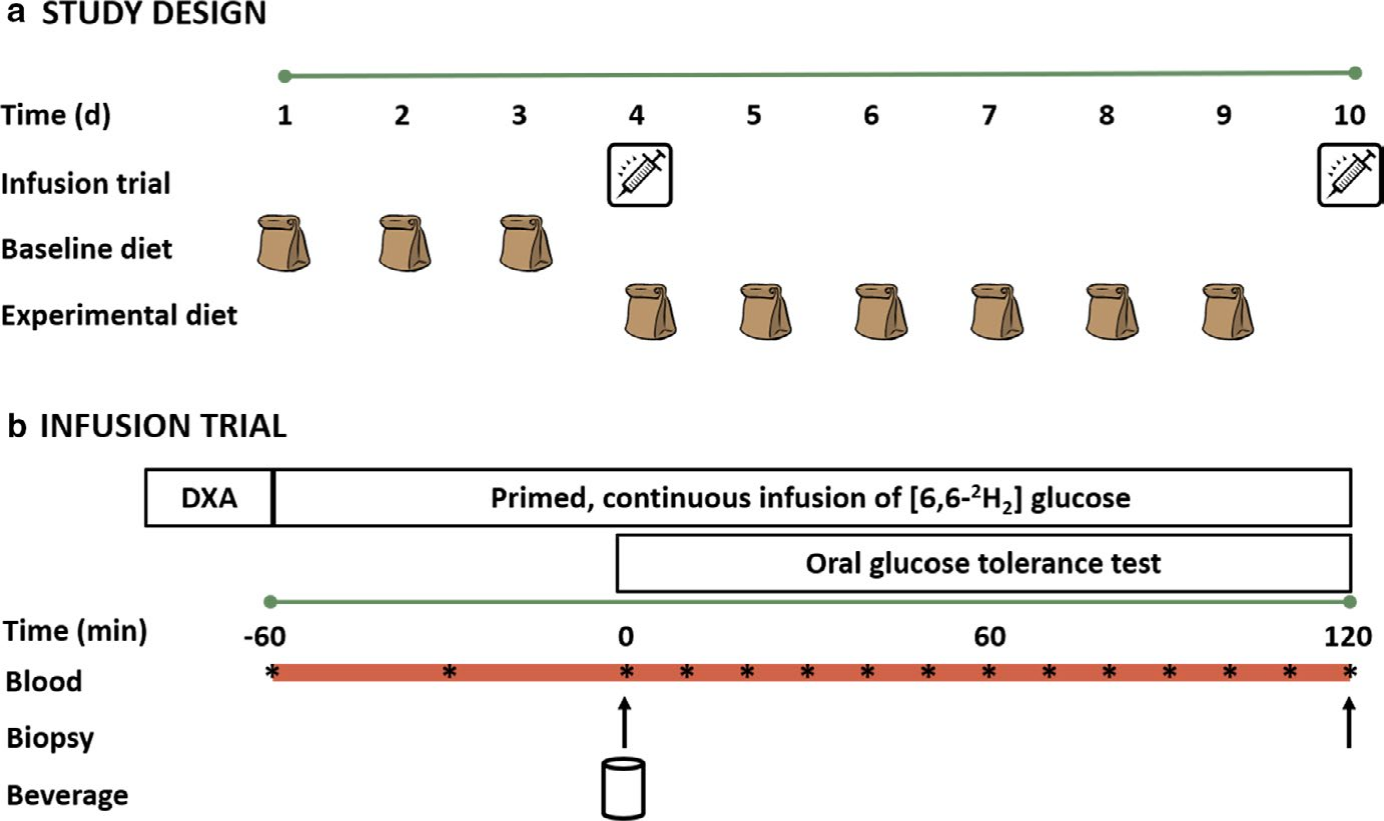
(a) Study design; and (b) infusion trial schematic. Both groups (high‐fat control [HF‐C] and high‐fat fish oil [HF‐FO]) completed all aspects depicted, with the only difference between groups being the n‐3PUFA fat content provided in the experimental diet. The beverage ingested in the infusion trial was a standard 75 g glucose in 300 ml water.

In a randomized, parallel study design, participants were assigned to either a high‐fat control (HF‐C; *n* = 10) or a high‐fat fish oil (HF‐FO; *n* = 10) group. Groups were matched based on age, body mass, habitual physical activity, and energy intake. Both groups were provided with a baseline diet replicating their habitual diet for 3d, followed by a high‐fat energy excess (HFEE) diet for 6d. Before and after the 6d HFEE diet, identical testing days were conducted that included infusion trials combined with an oral glucose tolerance test (OGTT, Figure 1).

Participants arrived at the laboratory between 06:00 and 08:00 h following a ~10h overnight fast. Dietary compliance was confirmed verbally prior to beginning the trial, nude body mass was recorded, and a dual‐energy x‐ray absorptiometry (DXA) scan (LUNAR iDXA; GE Healthcare Systems, UK) was performed to measure body composition. With participants positioned supine on a bed, a catheter was inserted into a forearm vein for collection of a basal venous blood sample followed by a primed (13.5 µmol/kg) continuous 3h infusion (0.350 µmol·kg^−1^·min^−1^) of [6,6‐^2^H_2_] glucose (Cambridge Isotope Laboratories, MA, USA) using an Alaris PK infusion pump (CareFusion, CA, USA). During the first hour of infusion, a second catheter was inserted into the contralateral arm for repeated venous blood sampling. A venous blood sample was obtained at –30 min (30 min into infusion but 30 min before beginning the OGTT). One hour into the infusion (0 min), participants consumed a test drink containing 73 g glucose + 2 g [U‐^13^C] labeled glucose in 300 ml water over a 5‐min period to start the OGTT. Fluid intake was restricted to only the OGTT drink throughout the trial. During the 2‐h period following OGTT drink ingestion, blood samples were drawn every 10 min until the continuous glucose infusion was stopped (2‐h post glucose OGTT drink consumption). On both trial days, muscle biopsies were collected before the OGTT (*i.e.,* in the basal state) to evaluate skeletal muscle phospholipid composition, skeletal muscle ceramide content, maximal activity of mitochondrial enzymes (citrate synthase and β‐hydroxyacyl‐CoA dehydrogenase), and kinase activity of AMPKα2 and PKB. Biopsies taken after the 2 h OGTT protocol (*i.e*., in the insulin‐stimulated state) were used to assess the kinase activity of PKB.

#### 2.2.1 Dietary assessment and composition

Food was provided to participants in daily food bags for a 9d period (3d baseline diet, 6d HFEE). As food was mostly consumed away from the laboratory, participants were instructed to solely consume the food and drink items provided during the experiment, and to consume the entirety of the food bag provided each day. All participants verbally confirmed consumption of their prescribed intake when collecting a new food bag.

Energy intake for the control and experimental diets was estimated from a 3d food (2 weekdays, 1 weekend day) and physical activity diary that participants completed prior to baseline testing. Food diaries were analyzed using commercially available dietary analysis software (Microdiet, Downlee Systems Ltd, UK) for average daily energy intake and macronutrient contributions. The 3d baseline diet was matched to participants’ habitual energy intake and macronutrient composition. The 6d high‐fat control (HF‐C) and high‐fat fish oil (HF‐FO) diets were consumed between the 2 testing days, and comprised 150% of habitual energy intake, with 60% of energy from fat, 25% from carbohydrate, and 15% from protein. The HF‐FO group had 10% of total fats in the diet (6% total energy) replaced by dietary fish oil, whereas HF‐C received the 60% fat from a mixture of sources, excluding foods highly enriched with n‐3PUFAs. Dietary fish oil was provided by means of oily fish consumption (salmon (1:2 ratio of EPA to DHA), mackerel (1:1 ratio of EPA to DHA), fish oil supplement (1:1 ratio of EPA to DHA) added to yoghurt‐based smoothies, and omega‐3 enriched juices containing 1,000mg EPA and 1,000mg DHA (Smartfish Nutrition, Norway). Placebo drinks (Smartfish Nutrition, Norway) without any omega‐3 oil were provided to participants in HF‐C.

### 2.3 Blood and skeletal muscle sampling and processing

Blood samples were drawn at baseline and every 10 min during the OGTT and dispensed into EDTA vacutainers (Becton, Dickinson & Company, NJ, USA). One basal blood sample was retained as whole blood for lipid analysis. All other blood samples were centrifuged at 3,500 rpm for 15 min at 4°C, the plasma removed, and then aliquoted into Eppendorf tubes for storage at −80°C pending analysis of glucose and insulin concentrations.

For the muscle biopsy, a 5‐mm Bergström needle was inserted through a pre‐prepared anaesthetized (2% Lidocaine) incision in the skin and fascia, into the muscle belly of the *vastus lateralis* and a muscle tissue sample was removed under manual suction. Muscle tissue was immediately rinsed using ice‐cold saline (0.9%) and any visible fat or connective tissue was removed. Samples were blotted, split into smaller pieces and weighed before freezing as aliquots in liquid nitrogen prior to storage at −80°C pending analysis. Both biopsies taken on the trial day (basal (0 min) and insulin‐stimulated (120 min)) were removed from the same leg, but from separate incision sites. For infusion trial 2 (post‐HFEE), biopsies were obtained from the contralateral limb.

### 2.4 Plasma glucose kinetics and concentrations, insulin concentrations and insulin sensitivity

#### 2.4.1 Plasma glucose and serum insulin analyses

Plasma glucose concentration was assessed spectrophotometrically using the glucose oxidase method on an ILab Aries benchtop analyzer (Instrumentation Laboratories, MA, USA). Samples were analyzed in duplicate for each 10 min sample interval during the OGTT (0–120 min). Serum insulin concentration was measured at the same time‐points by ELISA method according to manufacturer's instructions (Demeditic (DE2935), Germany).

#### 2.4.2 Plasma glucose kinetics

Plasma enrichments of [6,6‐^2^H_2_] glucose from the infusion and [U‐^13^C] glucose from the glucose drink were measured by GCMS following preparation of pentacetate derivatives using 2:1 acetic anhydride:pyridine (v/v). Plasma glucose enrichments were quantified using GCMS with chemical ionization. Ions were selectively monitored at mass to charge ratios of 331.1 (M + 0), 332.1 (M + 1), 333.1 (M + 2), 334.1 (M + 3), and 337.1 (M + 6) based on the masses of the labeled isotopes, and potential for carbon recycling. Plasma glucose enrichments for each ion were expressed relative to enrichments at 331.1 (M + 0). A skew correction factor was applied to the U‐^13^C enrichments (337.1; M + 6) to account for differences in the relative distribution of the mass spectra between the tracer (U‐^13^C) and tracee (unlabeled glucose). The GC System (7890A; Agilent Technologies, CA, USA) was interfaced with a 5975C MS inert XL EI/CI MSD with a triple‐axis detector. Methane gas was used for positive ionization, with helium as the carrier gas. Chemstation software (Agilent Technologies, CA, USA) was used for data analysis.

#### 2.4.3 Insulin sensitivity

Insulin sensitivity (or resistance) was estimated from the Matsuda Insulin Sensitivity Index (ISI; (Matsuda & DeFronzo, 1999)), HOMA‐IR (Matthews et al., 1985), HOMA‐β (Matthews et al., 1985), Hepatic Insulin Resistance (IR) index (Abdul‐Ghani et al. 2007), and Hepatic Insulin Sensitivity (IS) index (Faerch et al., 2010) using basal and insulin‐stimulated plasma glucose and serum insulin concentrations.

### 2.5 Blood and skeletal muscle lipid analysis

Fatty acid methyl esters (FAME) were extracted from whole blood lipids using the Rapid Omega Test, as previously described (Bell et al., 2011). Total lipids were extracted from skeletal muscle homogenates (Folch, Lees, & Sloane Stanley, 1957), with non‐lipid impurities removed by washing with 0.88% (w/v) KCl. Phospholipids were isolated using thin layer chromatography as described previously (Bell et al., 2011).

FAME from blood and skeletal muscle tissue were separated by GLC using a ThermoFisher Trace GC 2000 (ThermoFisher, Hemel Hempstead, UK) equipped with a fused silica capillary column (ZBWax, 60 m x 0.32 x 0.25 mm i.d.; Phenomenex, Macclesfield, UK). Hydrogen was used as carrier gas with on‐column injection. Individual methyl esters were identified from previously published data (Ackman et al., 1980) and the Chromcard for Windows (version 2.00) computer software (Thermoquest Italia S.p.A., Milan, Italy) was used for data collection and processing.

### 2.6 Skeletal muscle ceramide analyses

Total lipids were extracted from skeletal muscle homogenates (Folch et al., 1957) with non‐lipid impurities removed by washing with 0.88% (w/v) KCl. C17:0 ceramide (Avanti Polar Lipids Inc., Alabaster, AL, USA) was added as an internal standard. Ceramides were isolated using solid‐phase extraction chromatography (100 mg, 3 mL silica columns, Biotage, Uppsala, Sweden) and eluted in 5‐mL chloroform/ ethyl acetate (1:1) prior to quantification by liquid chromatography‐tandem mass spectrometry (LC‐MS/MS) as described previously (Mcilroy et al., 2016). All analyses were performed on a Thermo TSQ Quantum Ultra triple quadrupole mass spectrometer interfaced to a Thermo Accela 1250 UHPLC system in positive ion mode. Individual ceramide species were separated on a Kinetex C8 LC column (100 x 2.1mm x 2.6µm, Phenomenex, Macclesfield, UK) using an acetonitrile/ water gradient and detected through multiple reaction monitoring (MRM). The data were processed using Thermo Xcalibur 2.1 Quan Browser software.

### 2.7 Skeletal muscle mitochondrial enzyme assays

Skeletal muscle samples (5–12 mg) were homogenized in 100 µl of homogenizing solution per 1‐mg wet weight muscle (1.36 g of 0.1 M KH_2_PO_4_ + 50 mg of BSA in 80‐mL ddH_2_O; pH adjustment to 7.3 with KOH before adding additional ddH_2_O to achieve a final volume of 100 ml) using an automated homogenizer with 1.4‐mm (green) ceramide beads (MagNaLyser, Roche, Germany). Samples were homogenized on ice twice at 7,000 rpm for 10 s, separated by 1 min on ice. Following homogenization, all samples were centrifuged at 12266 *g* at 4°C for 20 s. Homogenates were snap‐frozen in liquid nitrogen before performing two freeze/ thaw cycles. Following the final thaw, homogenates (300 µl) were transferred into individual glass vials (cuvettes) for analysis and positioned in the sample rotor according to the work list schedule on an ILab Aries benchtop analyzer (Instrumentation Laboratories, MA, US). Twenty‐five microliters of muscle homogenate was used for each reaction. The work list schedule and homogenate sample were the same for both the citrate synthase and β‐HAD reactions.

### 2.8 Citrate synthase (CS)

Two‐hundred and twenty‐five microliters of reagent 1 (TRIS buffer (pH 8.3; 7.5 ml), DTNB (1.25 ml), acetyl‐CoA (2 ml), and Triton X‐100 (10%; 500 µl)) and the muscle homogenate (10 µl) were combined initially, before adding 15 µl of 10‐mM oxaloacetate (reagent 2) to start the reaction. Readings were taken at 450 nm at selected intervals (0, 39, 52, 65, 78, 91, 104, 130, 156, 182 195, 234, 260 s) over a 5‐min period.

### 2.9 β‐Hydroxyacyl‐CoA Dehydrogenase (β‐HAD)

Two‐hundred and fifteen microliters of reagent 1 (TRIS‐HCl buffer (pH 7.0; 2.5 ml), EDTA (0.5 ml), NADH (2.5 ml) and ddH_2_O (19.5 ml)) and 5 µl of reagent 2 (Triton x‐100 (10%)) were combined with the muscle homogenate (25 µl) for an initial 5‐min incubation period before taking a slope measurement at 340 nm for 2 min to record steady state. Next, 5 µl of 5‐mM acetoacetyl‐CoA (reagent 3) was added to start the reaction and the decrease in NADH was measured. Absorbance readings at 450 nm were obtained at regular intervals over a 2.5‐min period for determination of β‐HAD.

### 2.10 Skeletal muscle [γ‐^32^P] ATP kinase activity assays

PKB and AMPK activities were measured using methods previously described (McGlory et al., 2014).

### 2.11 Statistical analyses

All data were tested for normality using the Ryan Joiner test with Minitab software (version 16; Minitab, State College, PA). In the case of non‐normally distributed data, Box‐Cox transformations were applied before testing for normality a second time. A two‐way repeated measures ANOVA (IBM SPSS Statistics v19, NY, USA) was used to assess differences between groups [HF‐C versus HF‐FO] and over time [pre‐HFEE versus post‐HFEE]. In the case of a significant interaction effect, paired samples *t*‐tests were conducted to assess differences over time within each group alone. A two‐tailed Pearson correlation coefficient was used to assess the relationship between pre‐HFEE CS activity or β‐HAD activity and the pre‐ to post‐HFEE change in whole‐body indices of insulin sensitivity, skeletal muscle ceramide content, and skeletal muscle PKB and AMPKα2 activity. Statistical significance was assumed at the level of *p* < .05. All values are presented as mean ± standard deviation (*SD*), unless stated otherwise.

## 3 RESULTS

### 3.1 Participant characteristics, dietary intake and body composition

Participant characteristics for the HF‐C and HF‐FO groups on enrolment into the study are presented in Table 1. Dietary energy intake was increased by 150% (HF‐C: 10,937 ± 2,381 to 16,397 ± 3,573 kJ/d; HF‐FO: 10,222 ± 1,741 to 15,305 ± 2,690 kJ/d) with 60% of energy provided from fat. As intended, dietary n‐3PUFA intake was greater (*p* = .0001) in HF‐FO (25 ± 2 g) versus HF‐C (2 ± 0 g) (Table 2). Measurements of total body mass (HF‐FO: *p* = .003; HF‐C: *p* = .045) and total fat mass (HF‐FO: *p* = .016; HF‐C: *p* = .013) increased in response to HFEE, irrespective of group (Table 3), with significant gains in trunk fat mass (*p* = .033) and android fat (*p* = .001) observed in HF‐C, and significant gains in limb fat mass in HF‐FO (*p* = .015).

**Table 2:** Daily diet composition over 3 d of baseline diets and 6 d of experimental diets for the high‐fat control (HF‐C, *n* = 10) and high‐fat fish oil (HF‐FO, *n* = 10) groups.

	Baseline diets		Experimental diets	
	HF‐C	HF‐FO	HF‐C	HF‐FO
Energy (kJ/d)	10,937 ± 2,381	10,222 ± 1,741	16,397 ± 3,573^†^	15,305 ± 2,690^†^
Fat	104 ± 29	87 ± 15	261 ± 57^†^	245 ± 43^†^
SFA	42 ± 11	33 ± 10	106 ± 22^†^	100 ± 16^†^
MUFA	31 ± 11	24 ± 6	74 ± 14^†^	69 ± 14^†^
PUFA	10 ± 5	11 ± 6	31 ± 8^†^	38 ± 10^†^
*n*−3 PUFA	0 ± 0	0 ± 0	2 ± 0^†^	25 ± 6^†^ ^,^ *
Carbohydrate	323 ± 71	325 ± 105	263 ± 57^†^	244 ± 42^†^
Sugars	119 ± 39	133 ± 61	121 ± 22	77 ± 6^†^ ^,^ *
Protein	116 ± 41	108 ± 22	146 ± 33^†^	137 ± 24^†^

Note

Values are means ± *SD* and reported as g/d unless otherwise indicated. Values displayed as 0 were < 0.5g/d.

† Significantly different from baseline diet within each group.

* Significantly different from HF‐C for equivalent condition, *p* < .05.

**Table 3:** Body composition for high‐fat control (HF‐C, *n* = 10) and high‐fat fish oil (HF‐FO, *n* = 10) groups at baseline before (Pre‐HFEE), and following (Post‐HFEE) 6 days of high‐fat energy excess.

	HF‐C		HF‐FO	
	Pre‐HFEE	Post‐HFEE	Pre‐HFEE	Post‐HFEE
Total body mass (kg)	71.74 ± 11.38	72.25 ± 11.54^†^	69.65 ± 7.35	70.67 ± 7.24^†^
Total fat mass (kg)	13.87 ± 3.52	14.16 ± 3.43^†^	14.40 ± 5.23	14.85 ± 5.08^†^
Limb fat mass (kg)	6.94 ± 1.55	7.01 ± 1.56	7.40 ± 2.40	7.66 ± 2.42^†^
Trunk fat mass (kg)	6.07 ± 2.28	6.27 ± 2.21^†^	6.12 ± 2.85	6.29 ± 2.67
Android fat (%)	16.89 ± 7.54	17.88 ± 7.25^†^	17.63 ± 8.96	18.22 ± 8.62
Total lean mass (kg)	55.30 ± 9.58	55.55 ± 9.71	52.89 ± 6.25	53.44 ± 5.86

Note

Values are mean ± *SD*. No differences were observed between groups pre‐ or post‐HFEE.

^†^ significantly different from pre‐HFEE within group, *p* < .05.

### 3.2 Insulin sensitivity and plasma glucose kinetics

**Figure 2:**
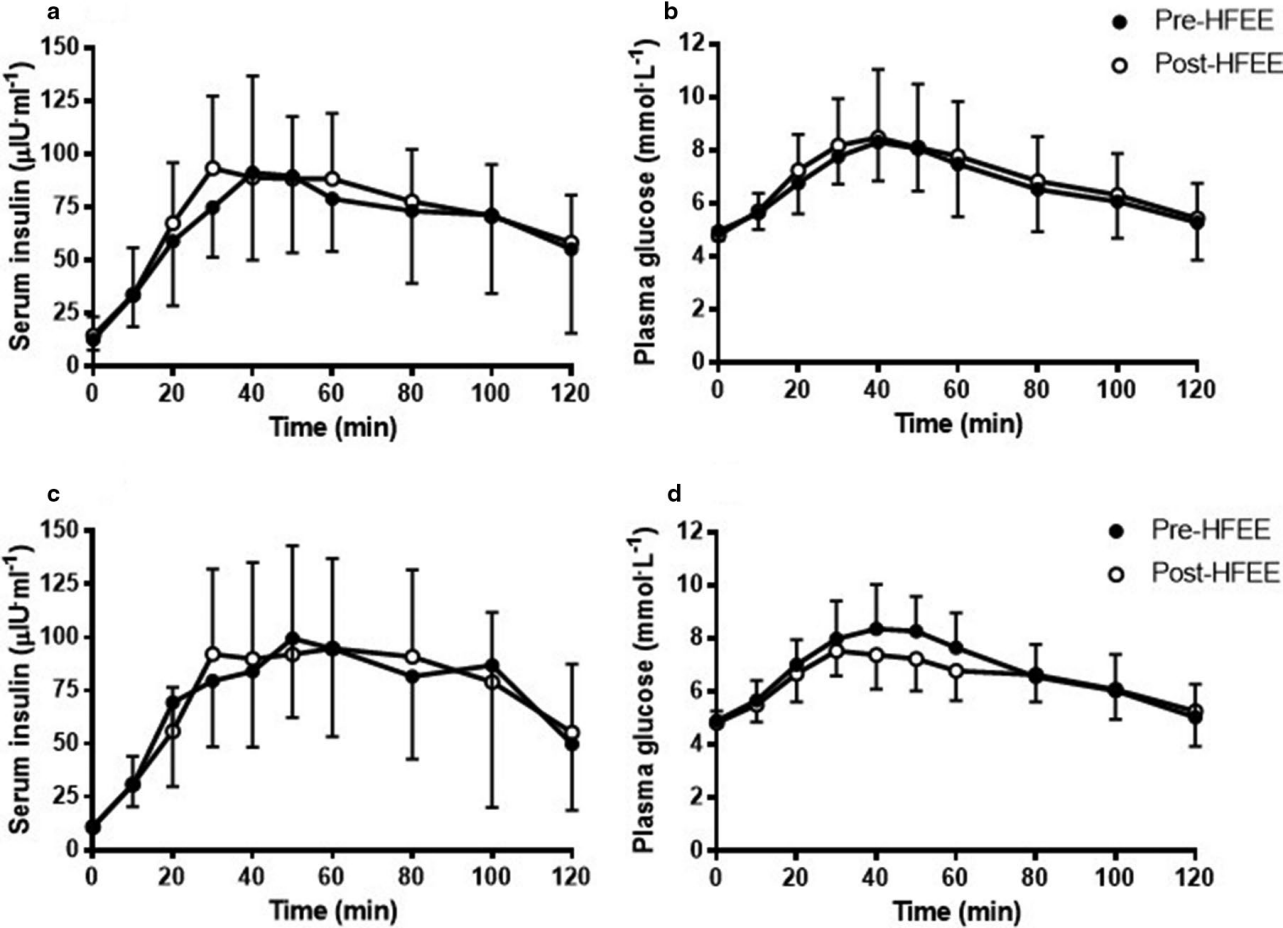
(a) Serum insulin and (b) plasma glucose concentration during the oral glucose tolerance test conducted before (Pre‐HFEE) and after (Post‐HFEE) high‐fat energy excess in the high‐fat control (HF‐C) group. (c) Serum insulin and (d) plasma glucose concentration during the oral glucose tolerance test conducted before (Pre‐HFEE) and after (Post‐HFEE) high‐fat energy excess in the high‐fat fish oil (HF‐FO) group. No significant trial (pre‐ to post‐HFEE), or interaction (trial by group) effects were noted.

Insulin sensitivity, assessed using Matsuda ISI, did not change following HFEE (HF‐C: 3.96 ± 1.85 to 3.59 ± 1.50, *p* = .480; HF‐FO: 3.81 ± 1.16 to 3.94 ± 1.09, *p* = .720) and was not different between groups. No difference was observed pre‐ to post‐HFEE when insulin sensitivity was assessed using HOMA‐IR, HOMA‐β, Hepatic‐IR index, and Hepatic‐IS index (data not shown). Insulin and glucose responses to the OGTT conducted pre‐ and post‐HFEE did not demonstrate any clear differences in glucose handling, nor between groups (Figure 2). No significant associations were observed between pre‐HFEE CS or β‐HAD activity and the pre‐ to post‐HFEE change in Matsuda ISI or HOMA‐IR.

Plasma glucose kinetics data, evaluated using dual‐glucose isotopic tracer analysis, are presented in Table 4. The AUC and peak plasma glucose concentrations for total Ra, exogenous Ra, endogenous Ra, and total Rd did not change following HFEE, irrespective of group. However, the time point at which exogenous glucose Ra peaked following oral glucose consumption was delayed following HFEE in the HF‐FO group only (*p* = .037).

**Table 4:** Plasma glucose kinetics data from baseline (Pre‐HFEE) and following 6 days of high‐fat energy excess (Post‐HFEE) in the high‐fat control (HF‐C, *n* = 10) and high‐fat fish oil (HF‐FO, *n* = 10) groups.

	HF‐C		HF‐FO	
	Pre‐HFEE	Post‐HFEE	Pre‐HFEE	Post‐HFEE
Glucose AUC_(0−120 min)_ (mg·min^−1^·kg^−1^:120 min)				
Total Ra	209.9 ± 67.9	195.6 ± 63.1	208.7 ± 79.9	213.3 ± 89.3
Exogenous Ra	318.2 ± 66.8	310.4 ± 46.4	349.9 ± 71.4	352.0 ± 88.1
Endogenous Ra	113.0 ± 26.0	118.4 ± 34.2	143.1 ± 39.8	139.0 ± 21.2
Total Rd	255.1 ± 89.0	278.2 ± 77.7	273.3 ± 90.7	283.2 ± 101.4
Peak glucose appearance/disappearance (mg·min^−1^·kg^−1^)				
Total Ra	6.47 ± 1.03	6.51 ± 0.82	7.11 ± 1.20	6.98 ± 1.36
Exogenous Ra	4.56 ± 0.94	4.81 ± 0.87	5.17 ± 1.12	5.34 ± 1.14
Endogenous Ra	3.55 ± 0.40	3.47 ± 0.34	3.77 ± 0.46	3.67 ± 0.29
Total Rd	6.69 ± 1.21	6.63 ± 0.89	7.34 ± 1.52	7.18 ± 1.58
Time point corresponding to peak plasma glucose appearance/disappearance (minutes)				
Total Ra	83 ± 29	95 ± 33	84 ± 30	100 ± 20
Exogenous Ra	105 ± 21	116 ± 13	95 ± 26	115 ± 13*
Endogenous Ra	15 ± 5	13 ± 5	13 ± 5	11 ± 3
Total Rd	77 ± 18	87 ± 25	85 ± 24	97 ± 19

Note

Values are means ± *SD*.

* Significantly different from Pre‐HFEE: main effect of time (*p* = .037) by ANOVA.

### 3.3 Lipid profiles

#### 3.3.1 Blood

**Figure 3:**
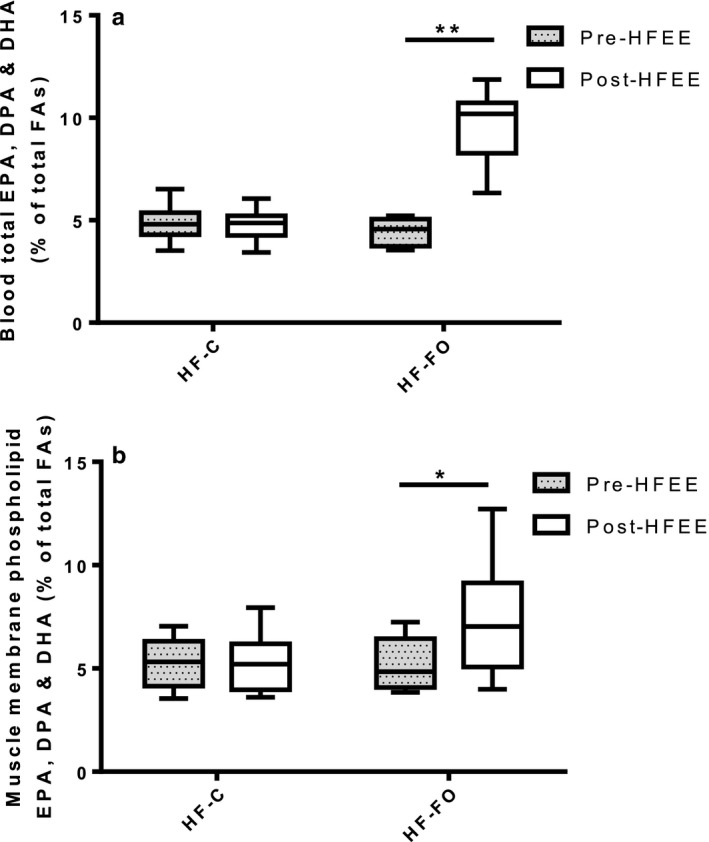
(a) Combined EPA, DPA, and DHA composition of whole blood pre‐ and post‐HFEE for both high‐fat control (HF‐C) and high‐fat fish oil (HF‐FO) groups (*n* = 10 per group); and (b) combined EPA, DPA and DHA composition of skeletal muscle phospholipid membrane fraction pre‐ and post‐HFEE for both HF‐C and HF‐FO groups (*n* = 9 per group). Data are expressed as median, 25th‐75th centile (box) and range (whiskers) and represent % of total fatty acids (FAs). ** significantly different *p* = .000002, and * significantly different *p* = .04, from pre‐HFEE in HF‐FO group only.

A full blood fatty acid profile is presented in Table S1. Total n‐3PUFAs (when expressed as a percentage of total FAs) were increased in HF‐FO (*p* < .0001) following HFEE, with no change in HF‐C (Figure 3a). An increased content of EPA (0.57 ± 0.40 to 3.95 ± 1.20%, *p* < .0001), DPA (1.40 ± 0.20 to 1.56 ± 0.14%, *p* = .0001), and DHA (2.51 ± 0.44 to 4.21 ± 0.58%, *p* < .0001) primarily contributed to the increase in total blood n‐3PUFAs observed in HF‐FO. The AA:EPA ratio, representative of the n‐6:n‐3 PUFA ratio, was decreased in HF‐FO over time (20.14 ± 4.98 to 3.01 ± 1.06, *p* < .0001), with no significant change in HF‐C (16.66 ± 3.71 to 15.23 ± 2.83, *p* = .0872).

#### 3.3.2 Skeletal muscle

A full skeletal muscle phospholipid profile is presented in Table S2. Total SFA, total MUFA, and total n‐6 PUFAs did not change following HFEE and were not different between groups. An increase in n‐3PUFA content in the phospholipid membrane of the HF‐FO group was observed following HFEE (Figure 3b, *p* = .040), with no change in HF‐C (*p* = .740). Specifically, EPA content was increased (*p* = .015), but AA was unchanged (*p* = .240) in the HF‐FO group following HFEE, with no changes in either within the HF‐C group. There was also no change in DHA content in HF‐FO (*p* = .260) or HF‐C (*p* = .740). The AA:EPA ratio decreased in the HF‐FO group (*p* = .037) over time with no change in HF‐C (*p* = .760).

### 3.4 Mitochondrial enzyme activities

Complete data for citrate synthase and β‐HAD activity were obtained from only *n* = 17 participants (*n* = 9 HF‐FO and *n* = 8 HF‐C) due to availability of muscle tissue. In the basal state (0 min), enzyme activities were not changed by HFEE in either group (Table 5). Mean pre‐HFEE activity of both mitochondrial enzymes were consistent with maximal activity values for moderately active individuals (Perry et al., 2007; Talanian et al., 2007); i.e. >20 µmol·g^‐1^·min^‐1^ for CS, and > 10 µmol·g^‐1^·min^‐1^ for β–HAD).

**Table 5:** Basal skeletal muscle mitochondrial enzyme maximal activity before (Pre‐HFEE) and after (Post‐HFEE) high‐fat energy excess for high‐fat control (HF‐C, *n* = 8) and high‐fat fish oil (HF‐FO, *n* = 9) groups.

	HF‐C		HF‐FO	
	Pre‐HFEE	Post‐HFEE	Pre‐HFEE	Post‐HFEE
Citrate synthase (µmol·g^−1^·min^−1^)	29.65 ± 9.41	27.51 ± 9.27	30.69 ± 5.70	27.78 ± 8.87
β‐HAD (µmol·g^−1^·min^−1^)	13.57 ± 3.94	14.44 ± 2.82	14.67 ± 1.87	14.07 ± 2.75

Note

Values are means ± *SD*. No significant differences were detected between groups or over time.

### 3.5 Skeletal muscle ceramides

**Figure 4:**
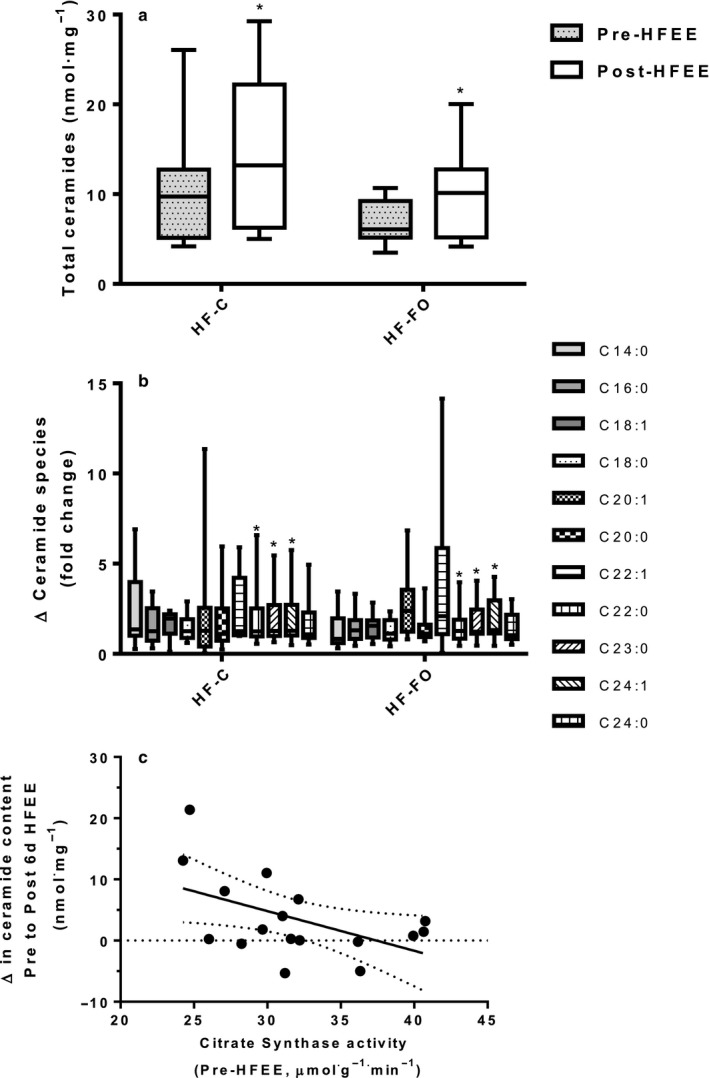
(a) Total skeletal muscle ceramide content pre‐ and post‐ 6 days of high‐fat energy excess (HFEE) in high‐fat control (HF‐C) and high‐fat fish oil (HF‐FO) conditions; (b) individual ceramide species, presented for HF‐C and HF‐FO groups, expressed as the fold‐change from pre‐ to post‐HFEE; and (c) association between muscle maximal citrate synthase activity measured before 6 days of high‐fat energy excess (HFEE) and the pre‐ to post‐HFEE absolute change in skeletal muscle total ceramide content (HF‐C and HF‐FO groups combined). Data are expressed as median, 25th‐75th centile (box) and range (whiskers). *n* = 9 per group. * significantly different from pre‐HFEE, *p* < .05.

Total basal skeletal muscle ceramide content increased by 69 ± 32% following HFEE, irrespective of group (Figure 4a). Statistical analysis of individual species revealed significant increases in the fold change for species C22:0, C23:0, and C24:1 (*p* = .033, .021, and .019, respectively) from pre‐ to post‐HFEE (Figure 4b), irrespective of group. An inverse relationship existed between basal CS activity and the increase in muscle ceramide content in response to 6 d of HFEE (r = −0.51, *p* = .037, Figure 4c).

### 3.6 PKB and AMPK α2 activity

**Figure 5:**
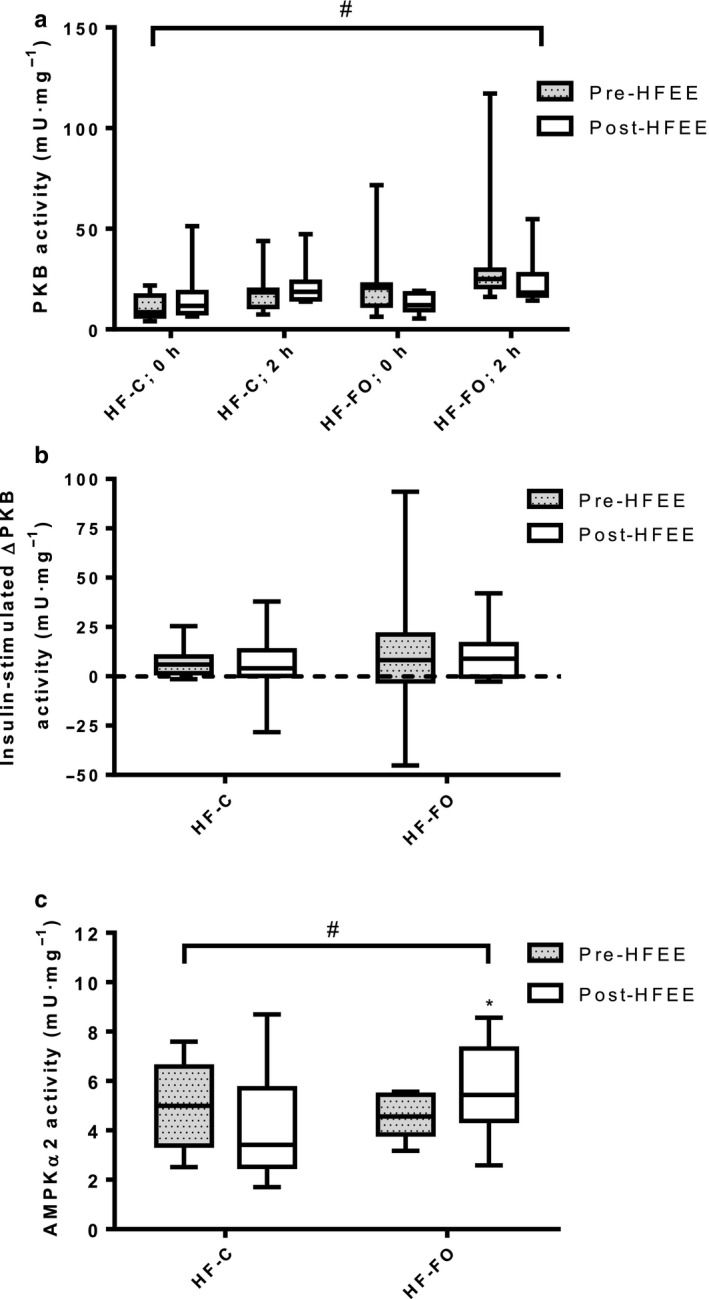
(a) PKB activity in the basal (0 hr) and insulin stimulated states (2 hr) pre‐ and post‐ 6 days of high‐fat energy excess (HFEE) for both high‐fat control (HF‐C) and high‐fat fish oil (HF‐FO) groups; (b) Insulin‐stimulated change in PKB activity for HF‐C and HF‐FO, pre‐ and post‐HFEE; and (c) Basal skeletal muscle AMPKα2 activity pre‐ and post‐HFEE for both HF‐C and HF‐FO groups. Data are expressed as median, 25th‐75th centile (box) and range (whiskers). *n* = 9 per group. # group by time interaction (pre‐ to post‐HFEE) for PKB and AMPKα2 activity, *p* = .025 and *p* = .038, respectively. * AMPKα2 activity significantly higher at post‐HFEE compared to pre‐HFEE in HF‐FO group only (*p* = .049).

PKB activity was greater in the insulin‐stimulated state (2 hr) than basal state (0 hr), irrespective of group, but did not reach statistical significance (*p* = .051; Figure 5a). However, a significant time‐by‐group interaction (*p* = .025) was observed, with PKB activity decreasing in HF‐FO but increasing in HF‐C from pre‐ to post‐HFEE (mean difference (95% CI) +4.5 (−2.0 to +10.9) mU^.^mg^‐1^ for HF‐C and −10.1 (−20.4 to + 0.2) mU^.^mg^‐1^ for HF‐FO, Figure 5a). No difference in the change in PKB activity following insulin stimulation was observed between groups (Figure 5b). No association was observed between pre‐HFEE CS activity and the change in PKB activity pre‐ to post‐HFEE in either group (data not shown).

Basal (0 min) skeletal muscle AMPKα2 activity was unchanged following HFEE in HF‐C, but increased in HF‐FO (mean difference (95% CI) −0.8 (−2.3 to + 0.8) mU^.^mg^‐1^ for HF‐C and + 1.2 (+0.9 to +2.3) mU^.^mg^‐1^ for HF‐FO, Figure 5c; interaction effect: *p* = .038). No association was observed between pre‐HFEE CS activity and the change in AMPKα2 activity pre‐ to post‐HFEE (r = 0.112, *p* = .690).

## 4 DISCUSSION

We report physiologically important early signs of metabolic disturbance in skeletal muscle of moderately active individuals fed a HFEE diet for 6 d. Skeletal muscle ceramide content was increased following HFEE, irrespective of whether 10% of dietary fat was derived from n‐3PUFAs. The increase in ceramide content was inversely associated with skeletal muscle oxidative capacity, as determined by the maximal activity of citrate synthase. However, despite modest increases in ceramide content, no changes were noted in indices of whole‐body insulin sensitivity, or in glucose disposal/ kinetics. Differential responses in basal intramuscular PKB and AMPKα2 activity between HF‐C and HF‐FO suggest that the lipid composition of HFEE diet impacts upon early metabolic disturbances in cellular signaling pathways. Our findings provide insight into early localized (skeletal muscle) cellular changes in response to HFEE and provide the basis for more research investigating the potential for n‐3PUFAs to positively modulate intracellular signaling responses during whole‐body exposure to caloric excess.

### 4.1 Dietary intake and skeletal muscle lipid composition

Skeletal muscle ceramide content is closely linked with insulin resistance in skeletal muscle (Blachnio‐Zabielska et al., 2010) and liver (Zabielski et al., 2019). In the present study, the ceramide content of skeletal muscle increased over the 6 d of HFEE in both groups. Ceramide synthesis is complex with de novo synthesis resulting from a range of ceramide synthases that catalyze the formation of ceramide species from long chain fatty acids (Hannun & Obeid, 2008). An abundance of dietary lipids, in excess of capacity for oxidation, leads to a surplus of the ceramide precursor fatty acyl‐CoAs and subsequent upregulation of ceramide synthesis. Indeed, we observed an inverse association between the oxidative capacity of skeletal muscle and the increase in ceramide content in skeletal muscle. Our observations highlight that, in men who are moderately active, the muscle capacity for lipid oxidation is an important factor in the metabolic response to HFEE. Our data suggest that higher basal mitochondrial oxidative capacity may provide some protection against increases in ceramide content when exposed to HFEE diets. This observation is consistent with the conclusions of prior work demonstrating regular habitual physical activity can prevent pathophysiological outcomes associated with diet‐induced increases in body mass (Krogh‐Madsen et al., 2014).

Our data support findings from previous literature (Dzamko & Steinberg, 2009; Turner et al., 2007) by demonstrating that short‐term (6 d) hyper‐caloric diets do not drive changes in mitochondrial biogenesis in humans. Interestingly, although skeletal muscle ceramide content increased, there were no differences in ceramide accumulation between HF‐C and HF‐FO groups. This lack of difference between groups likely reflects a similar imbalance between ceramide synthesis and metabolism in both of our HFEE situations. In the present study, the main ceramide species that were elevated in response to HFEE were long chain ceramides (C22:0, C23:0, and C24:1). Long chain ceramide synthesis arises from the activity of ceramide synthase 2 which shows remarkable specificity to longer chain (C20‐C26) acyl‐CoAs (Laviad et al., 2008). Unfortunately, we were unable to examine the specific cellular locations of these ceramide species to identify potential differences between treatment groups. However, it is noteworthy that an increase in ceramide synthesis has been proposed to alter the activity of many different kinases (Schubert et al., 2000). Therefore, increases in ceramide synthesis would be expected to impact upon cellular signaling pathways in the context of HFEE, as modeled in the present study.

### 4.2 Dietary intake, glucose disposal and cellular signaling

Despite the increases in skeletal muscle ceramide content with HFEE, we failed to detect a difference in whole‐body glucose disposal or glucose kinetics during the OGTTs. While other studies have demonstrated an effect of short‐term high‐fat diets on glucose handling (Bachmann et al., 2001; Hulston et al., 2015; Parry et al., 2017, 2019), this outcome is not consistent across all studies (Adochio et al., 2009; Bakker et al., 2014; Brøns et al., 2009; Cornier et al., 2006). Studies reporting perturbed glucose handling following HFEE were of similar duration (6–7 d), and similar energy surplus (~50%) and fat content (~60%–65%) to the present study. However, These studies recruited a range of physically active participants (Hulston et al., 2015; Parry et al., 2017, 2019), used mixed‐sex participant groups (Hulston et al., 2015; Parry et al., 2017, 2019), and, in some instances, calculated energy provision from estimated energy requirements rather than from food / activity diary records (Parry et al., 2017, 2019). Thus, several methodological aspects may contribute to the differences in findings between studies (notably mixed‐sex samples), which should be considered in the design of future studies that utilize short‐term HFEE as a model to investigate early responses to overfeeding.

The absence of a difference in glucose handling over 6‐d HFEE may reflect the specific ceramide species affected by our HFEE diets. Typically, C16 or C18 ceramides are associated most strongly with changes in tissue insulin sensitivity (Straczkowski et al., 2004), and PKB activity would be expected to be downregulated by increases in C16 or C18 ceramides. In the present study, PKB activity was differentially regulated between treatment groups with an interaction effect for PKB activity. In the absence of significant increases in the C16 and C18 ceramide species, we would not expect to observe a downregulation of PKB activity by HFEE. However, an apparent downregulation of PKB kinase activity was evident in the HF‐FO group despite no difference in C16 and C18 ceramide content. This downregulation of PKB kinase in the HF‐FO group could reflect a positive change in the responsiveness/ efficiency of the intracellular signaling cascade. This hypothesis appears consistent with the observation of similar glucose handling and glucose kinetics observed during the OGTT, despite reduced basal and post‐OGTT PKB activity in the HF‐FO group. Alternatively, it could be interpreted that greater PKB activity was required in the HF‐C group to achieve the same level of glucose control. Either way, the differential response in PKB activity between HF‐C and HF‐FO from pre‐ to post‐HFEE most likely reflects differences in skeletal muscle phospholipid membrane incorporation of n‐3PUFAs, and/ or subsequent changes in skeletal muscle n‐6:n‐3 ratio. Supporting this notion, our previous work (McGlory et al., 2016) revealed an alteration in the efficiency of the signaling cascade following 8 wk of n‐3PUFA supplementation, and demonstrated a reduced skeletal muscle intracellular signaling response (*i.e.,* mTORC1 and p70S6k) to exogenous amino acid availability without a concomitant decrease in the rate of skeletal muscle protein synthesis. Furthermore, an attenuation of PDK activity was noted by Turvey et al., (2005) without any change in PDHa activity following n‐3PUFA ingestion as part of a HFEE diet. Therefore, n‐3PUFA ingestion seems to dampen, or introduce efficiency into, signaling cascades without compromising functional outcomes.

AMPKα2 activity also was differentially regulated between our two diet groups, with an increased basal AMPK activity noted in the HF‐FO group only. An increase in basal PKB activity can inhibit AMPK activity through the phosphorylation of serine 487 on the α1 subunit (Hardie, 2014). However, PKB does not phosphorylate the α2 subunit (Hawley et al., 2014), so a reduction in PKB activity would typically not be associated with an increase in AMPKα2 activity. In the present study, the differential response with increased basal AMPKα2 activity in the HF‐FO group may reflect an alteration in protein kinase C (PKC) signaling via diacylglycerol (DAG) accumulation. Unfortunately, we cannot confirm this hypothesis from the data available. An elevation in AMPK activity would typically be associated with cellular energy stress and promote an increased fatty acid uptake and oxidation, and reduction in fatty acid synthesis (Hardie, 2014). This energy stress responsiveness of AMPK highlights the central role that AMPK plays in lipid metabolism. The increased basal activity of AMPK in our HFEE situation was only evident in the HF‐FO group, suggesting that increasing n‐3PUFA consumption may offset some of the negative metabolic effects of HFEE diets. Notably, an increase in AMPK activity during energy surplus could be considered a positive outcome. In this regard, an increased activity of AMPK would reduce inflammatory responses through actions on nuclear factor kappa‐light‐chain‐enhancer of activated B cells (NF‐kB; (Salminen et al., 2011)), and AMPK activators such as metformin are beneficial for improving glucose control (Rena et al., 2017). However, further analysis of downstream targets, such as ACC2 phosphorylation status, would be required to confirm whether a biological action resulted from the differential regulation of AMPKα2 activity. Thus, increased activity of AMPK in response to HF‐FO diets requires further exploration, especially since energy excess would typically be associated with a downregulation of AMPK (Coughlan et al., 2013).

Finally, previous work by McGlory et al., (2014); McGlory et al. (2016) and Smith et al. (2011) have reported changes in skeletal muscle lipid membrane composition over 4–8 weeks with EPA rich fish oil supplementation. Smith et al. (2011) reported an increase in total n‐3PUFA content of the muscle lipid membrane fraction: increasing from 5.04% to 9.03% of total lipids after 8 weeks of ingesting 1.86 g EPA and 1.50 g DHA per day. McGlory et al. (2016) reported an increase in this same lipid membrane fraction from 5.53% to 11.16% after 8 weeks of supplementation with 3.50 g EPA and 0.90 g DHA per day. The present data indicate that modest changes in skeletal muscle lipid membrane composition (5.73 to 7.79%) can occur in only 6 d when 25 g of fish oil‐derived EPA/ DHA are included in a HFEE diet. Failure to perturb insulin sensitivity following short‐term energy‐excess in HF‐C meant that we were unable to cleanly evaluate the potential positive effects of dietary fish oil in the present study. However, membrane lipid composition changes are likely to influence the muscle responsiveness to nutritional and/ or contractile stimuli, and therefore are important in determining the cellular and metabolic outcomes induced by HFEE (Casares et al., 2019). It is worth noting that in the present study, EPA was elevated in the muscle phospholipid fraction in the HF‐FO group, and although AA was unchanged, these changes in lipid composition appear to drive metabolic benefits in skeletal muscle cells (Jeromson et al., 2018).

### 4.3 Study limitations

It is important to acknowledge that there are several limitations to our study that preclude us from making firm conclusions. Firstly, we chose a short‐term high HFEE diet to mimic short periods of overfeeding. Moreover, we replicated other short‐term overfeeding studies to assess the potential influence of lipid composition during HFEE on our primary outcomes (i.e., glucose handling), and to simulate typical dietary behaviors during seasonal holiday periods, for example, Christmas. In our study cohort, this methodological approach did not induce large metabolic alterations in glucose handling possibly because of methodological differences between our study and other studies, such as: activity status of participants; single sex group design; or, methods for assessing and prescribing HFEE diets. Secondly, we opted to substitute 10% of all fats with fish oil acutely, rather than use a lower priming dose of fish oil over several weeks before the high‐fat overfeeding period. Although no side effects of this high fish oil dose were reported, we acknowledge that this n‐3PUFA dosing strategy is not sustainable, or clinically applicable, as longer term use at this dose would not be recommended. Thirdly, we did not assess PKB activity under peak insulin stimulation but rather following the peak, with insulin being around 4–5 fold higher than basal concentrations at time of biopsy. Finally, due to the fairly small sample size in each group, and the repeated measures independent group design, there is a chance that some observations may reflect false‐positive outcomes that require replication before firm conclusions can be made.

### 4.4 Conclusions

In conclusion, the present data confirm previous observations that subcellular events resulting from HFEE precede the clear development of whole‐body insulin resistance. Six days of HFEE led to increased skeletal muscle ceramide content, particularly the longer chain ceramide species. Basal mitochondrial enzyme activities remained unchanged by HFEE in both HF‐FO and HF‐C groups, but skeletal muscle oxidative capacity was inversely associated with the fold change in skeletal muscle ceramide content, suggesting that physical activity status influences the response. The differentially altered basal AMPKα2 activity and PKB activity following HFEE in HF‐FO, compared to HF‐C, suggests early cellular/ metabolic advantages of n‐3PUFAs that could prove important during longer term periods of HFEE. It is interesting to speculate that the group divergence in these findings is a result of differences in the phospholipid membrane composition following n‐3PUFA ingestion. Alterations in the docking efficiency of transporter proteins in the plasma membrane, altered nutrient kinetics, and/ or signaling resulting from membrane composition changes all may contribute to the observed differences in kinase activity between groups. Further work is required to elucidate the mechanisms of lipid composition in the high‐fat overfed state, as well as the time course, and cellular locations, of changes in ceramide species during prolonged HFEE diets.

## CONFLICT OF INTEREST

The authors have no conflicts of interest. The Diabetes Research and Wellness Foundation supported the study with a grant to KDT, CM and SDRG. Fish oils were provided for the study by SMARTFISH Nutrition, Norway. SLW was supported by a University of Stirling funded PhD studentship.

## AUTHOR CONTRIBUTIONS

SLW, KDT, CNM, and SDRG designed the study. SLW, LSM, CM and OCW organized and carried out the clinical experiments. JRD, PDW, AAF, RRW, IYK, DLH, and SDRG supported the range of tissue sample analyses. SLW, SDRG, OCW, and CNM performed the statistical analyses and wrote the manuscript together with CM, KDT, and DLH. All authors have read and approved the final manuscript.

## Supporting information



Table S1‐S2

## References

[phy214529-bib-0001] Abdul‐Ghani, M. A. , Matsuda, M. , Balas, B. , & DeFronzo, R. A. (2007). Muscle and liver insulin resistance indexes derived from the oral glucose tolerance test. Diabetes Care, 30, 89–94. 10.2337/dc06-1519 17192339

[phy214529-bib-0002] Ackman, R. G. , Eaton, C. A. , & Dyerberg, J. (1980). Marine docosenoic acid isomer distribution in the plasma of Greenland Eskimos. American Journal of Clinical Nutrition, 33, l814–l817. 7190776 10.1093/ajcn/33.8.1814

[phy214529-bib-0003] Adams, J. M. , Pratipanawatr, T. , Berria, R. , Wang, E. , DeFronzo, R. A. , Sullards, M. C. , & Mandarino, L. J. (2004). Ceramide content is increased in skeletal muscle from obese insulin‐resistant humans. Diabetes, 53, 25–31. 10.2337/diabetes.53.1.25 14693694

[phy214529-bib-0004] Adochio, R. L. , Leitner, J. W. , Gray, K. , Draznin, B. , & Cornier, M.‐A. (2009). Early responses of insulin signaling to high‐carbohydrate and high‐fat overfeeding. Nutr Metab (Lond), 6, 37 10.1186/1743-7075-6-37 19781106 PMC2761378

[phy214529-bib-0005] Albert, B. B. , Derraik, J. G. B. , Brennan, C. M. , Biggs, J. B. , Smith, G. C. , Garg, M. L. , … Cutfield, W. S. (2014). Higher omega‐3 index is associated with increased insulin sensitivity and more favourable metabolic profile in middle‐aged overweight men. Scientific Reports, 4(1); 10.1038/srep06697. PMC5381193 25331725

[phy214529-bib-0006] Bachmann, O. P. , Dahl, D. B. , Brechtel, K. , Machann, J. , Haap, M. , Maier, T. , … Jacob, S. (2001). Effects of intravenous and dietary lipid challenge on intramyocellular lipid content and the relation with insulin sensitivity in humans. Diabetes, 50, 2579–2584. 10.2337/diabetes.50.11.2579 11679437

[phy214529-bib-0007] Bakker, L. E. H. , van Schinkel, L. D. , Guigas, B. , Streefland, T. C. M. , Jonker, J. T. , van Klinken, J. B. , … Jazet, I. M. (2014). A 5‐day high‐fat, high‐calorie diet impairs insulin sensitivity in healthy, young South Asian men but not in Caucasian men. Diabetes, 63, 248–258. 10.2337/db13-0696 24357702

[phy214529-bib-0008] Bell, J. G. , Mackinlay, E. E. , Dick, J. R. , Younger, I. , Lands, B. , & Gilhooly, T. (2011). Using a fingertip whole blood sample for rapid fatty acid measurement: Method validation and correlation with erythrocyte polar lipid compositions in UK subjects. British Journal of Nutrition, 106, 1408–1415. 10.1017/S0007114511001978 21736805

[phy214529-bib-0009] Blachnio‐Zabielska, A. , Baranowski, M. , Zabielski, P. , & Gorski, J. (2010). Effect of high fat diet enriched with unsaturated and diet rich in saturated fatty acids on sphingolipid metabolism in rat skeletal muscle. Journal of Cellular Physiology, 225, 786–791. 10.1002/jcp.22283 20568228

[phy214529-bib-0010] Borkman, M. , Storlien, L. H. , Pan, D. A. , Jenkins, A. B. , Chisholm, D. J. , & Campbell, L. V. (1993). The relation between insulin sensitivity and the fatty‐acid composition of skeletal‐muscle phospholipids. New England Journal of Medicine, 328, 238–244. 10.1056/NEJM199301283280404 8418404

[phy214529-bib-0011] Brøns, C. , Jensen, C. B. , Storgaard, H. , Hiscock, N. J. , White, A. , Appel, J. S. , … Vaag, A. (2009). Impact of short‐term high‐fat feeding on glucose and insulin metabolism in young healthy men. Journal of Physiology, 587, 2387–2397. 10.1113/jphysiol.2009.169078 19332493 PMC2697306

[phy214529-bib-0012] Casares, D. , Escribá, P. V. , Rosselló, C. A. , Casares, D. , Escribá, P. V. , & Rosselló, C. A. (2019). Membrane lipid composition: Effect on membrane and organelle structure, function and compartmentalization and therapeutic avenues. International Journal of Molecular Sciences, 20, 2167 10.3390/ijms20092167 PMC6540057 31052427

[phy214529-bib-0013] Chan, J. M. , Rimm, E. B. , Colditz, G. A. , Stampfer, M. J. , & Willett, W. C. (1994). Obesity, fat distribution, and weight gain as risk factors for clinical diabetes in men. Diabetes Care, 17, 961–969. 10.2337/diacare.17.9.961 7988316

[phy214529-bib-0014] Colditz, G. A. , Willett, W. C. , Rotnitzky, A. , & Manson, J. E. (1995). Weight gain as a risk factor for clinical diabetes mellitus in women. Annals of Internal Medicine, 122, 481–486. 10.7326/0003-4819-122-7-199504010-00001 7872581

[phy214529-bib-0015] Cornier, M.‐A. , Bergman, B. C. , & Bessesen, D. H. (2006). The effects of short‐term overfeeding on insulin action in lean and reduced‐obese individuals. Metabolism, 55, 1207–1214. 16919540 10.1016/j.metabol.2006.05.003

[phy214529-bib-0016] Coughlan, K. A. , Valentine, R. J. , Ruderman, N. B. , & Saha, A. K. (2013). Nutrient excess in AMPK downregulation and insulin resistance. Journal of Endocrinology, Diabetes & Obesity, 1, 1008. PMC4479300 26120590

[phy214529-bib-0017] Dzamko, N. L. , & Steinberg, G. R. (2009). AMPK‐dependent hormonal regulation of whole‐body energy metabolism. Acta Psychologica, 196, 115–127. 10.1111/j.1748-1716.2009.01969.x 19245657

[phy214529-bib-0018] Faerch, K. , Brøns, C. , Alibegovic, A. C. , & Vaag, A. (2010). The disposition index: Adjustment for peripheral vs. hepatic insulin sensitivity? Journal of Physiology, 588, 759–764. 20100741 10.1113/jphysiol.2009.184028 PMC2834935

[phy214529-bib-0019] Fedor, D. , & Kelley, D. S. (2009). Prevention of insulin resistance by n‐3 polyunsaturated fatty acids. Current Opinion in Clinical Nutrition and Metabolic Care, 12, 138–146. 10.1097/MCO.0b013e3283218299 19202385

[phy214529-bib-0020] Folch, J. , Lees, M. , & Sloane Stanley, G. H. (1957). A simple method for the isolation and purification of total lipides from animal tissues. Journal of Biological Chemistry, 226, 497–509. 13428781

[phy214529-bib-0021] Hancock, C. R. , Han, D.‐H. , Chen, M. , Terada, S. , Yasuda, T. , Wright, D. C. , & Holloszy, J. O. (2008). High‐fat diets cause insulin resistance despite an increase in muscle mitochondria. Proceedings of the National Academy of Sciences, 105, 7815–7820. 10.1073/pnas.0802057105 PMC2409421 18509063

[phy214529-bib-0022] Hannun, Y. A. , & Obeid, L. M. (2008). Principles of bioactive lipid signalling: Lessons from sphingolipids. Nature Reviews Molecular Cell Biology, 9, 139–150. 10.1038/nrm2329 18216770

[phy214529-bib-0023] Hardie, D. G. (2014). AMPK ‐ Sensing energy while talking to other signaling pathways. Cell Metabolism, 20, 939–952. 10.1016/j.cmet.2014.09.013 25448702 PMC5693325

[phy214529-bib-0024] Harris, W. S. (1996). n‐3 fatty acids and lipoproteins: Comparison of results from human and animal studies. Lipids, 31, 243–252. 10.1007/BF02529870 8900453

[phy214529-bib-0025] Harris, W. S. (1997). n‐3 fatty acids and serum lipoproteins: Human studies. American Journal of Clinical Nutrition, 65, 1645S–1654S. 10.1093/ajcn/65.5.1645S 9129504

[phy214529-bib-0026] Hawley, S. A. , Ross, F. A. , Gowans, G. J. , Tibarewal, P. , Leslie, N. R. , & Hardie, D. G. (2014). Phosphorylation by Akt within the ST loop of AMPK‐α1 down‐regulates its activation in tumour cells. The Biochemical Journal, 459, 275–287. 10.1042/BJ20131344 24467442 PMC4052680

[phy214529-bib-0027] Holland, W. , Knotts, T. , Chavez, J. , Wang, L. , Hoehn, K. , & Summers, S. (2007). Lipid mediators of insulin resistance. Nutrition Reviews, 65, 39–46. 10.1301/nr.2007.jun.S39-S46 17605313

[phy214529-bib-0028] Holloway, G. P. , Bonen, A. , & Spriet, L. L. (2009). Regulation of skeletal muscle mitochondrial fatty acid metabolism in lean and obese individuals. American Journal of Clinical Nutrition, 89, 455S–S462. 19056573 10.3945/ajcn.2008.26717B

[phy214529-bib-0029] Hulston, C. J. , Churnside, A. A. , & Venables, M. C., (2015). Probiotic supplementation prevents high‐fat, overfeeding‐induced insulin resistance in human subjects. Br J Nutr1–7. 10.1017/S0007114514004097 PMC4339038 25630516

[phy214529-bib-0030] Jain, S. S. , Paglialunga, S. , Vigna, C. , Ludzki, A. , Herbst, E. A. , Lally, J. S. , … Holloway, G. P. (2014). High‐fat diet‐induced mitochondrial biogenesis is regulated by mitochondrial‐derived reactive oxygen species activation of CaMKII. Diabetes, 63, 1907–1913. 10.2337/db13-0816 24520120

[phy214529-bib-0031] Jeromson, S. , Mackenzie, I. , Doherty, M. K. , Whitfield, P. D. , Bell, G. , Dick, J. , … Hamilton, D. L. (2018). Lipid remodeling and an altered membrane‐associated proteome may drive the differential effects of EPA and DHA treatment on skeletal muscle glucose uptake and protein accretion. American Journal of Physiology‐Endocrinology and Metabolism, 314, E605–E619. 10.1152/ajpendo.00438.2015 28655718

[phy214529-bib-0032] Krogh‐Madsen, R. , Pedersen, M. , Solomon, T. P. J. , Knudsen, S. H. , Hansen, L. S. , Karstoft, K. , … Pedersen, B. K. (2014). Normal physical activity obliterates the deleterious effects of a high‐caloric intake. Journal of Applied Physiology, 116, 231–239. 10.1152/japplphysiol.00155.2013 24201706

[phy214529-bib-0033] Laviad, E. L. , Albee, L. , Pankova‐Kholmyansky, I. , Epstein, S. , Park, H. , Merrill, A. H. , & Futerman, A. H. (2008). Characterization of ceramide synthase 2: Tissue distribution, substrate specificity, and inhibition by sphingosine 1‐phosphate. Journal of Biological Chemistry, 283, 5677–5684. 10.1074/jbc.M707386200 18165233

[phy214529-bib-0034] Matsuda, M. , & DeFronzo, R. A. (1999). Insulin sensitivity indices obtained from oral glucose tolerance testing: Comparison with the euglycemic insulin clamp. Diabetes Care, 22, 1462–1470. 10.2337/diacare.22.9.1462 10480510

[phy214529-bib-0035] Matthews, D. R. , Hosker, J. P. , Rudenski, A. S. , Naylor, B. A. , Treacher, D. F. , & Turner, R. C. (1985). Homeostasis model assessment: Insulin resistance and beta‐cell function from fasting plasma glucose and insulin concentrations in man. Diabetologia, 28, 412–419. 3899825 10.1007/BF00280883

[phy214529-bib-0036] McGlory, C. , Galloway, S. D. R. , Hamilton, D. L. , McClintock, C. , Breen, L. , Dick, J. R. , … Tipton, K. D. (2014). Temporal changes in human skeletal muscle and blood lipid composition with fish oil supplementation. Prostaglandins Leukotrienes and Essential Fatty Acids, 90, 199–206. 10.1016/j.plefa.2014.03.001 24726616

[phy214529-bib-0037] McGlory, C. , Wardle, S. L. , Macnaughton, L. S. , Witard, O. C. , Scott, F. , Dick, J. , … Tipton, K. D. (2016). Fish oil supplementation suppresses resistance exercise and feeding‐induced increases in anabolic signaling without affecting myofibrillar protein synthesis in young men. Physiological Reports, 4(6), e12715 10.14814/phy2.12715 27009278 PMC4814892

[phy214529-bib-0038] McGlory, C. , White, A. , Treins, C. , Drust, B. , Close, G. L. , MacLaren, D. P. M. , … Hamilton, D. L. (2014). Application of the [γ‐ ^32^ P] ATP kinase assay to study anabolic signaling in human skeletal muscle. Journal of Applied Physiology, 116, 504–513. 24436296 10.1152/japplphysiol.01072.2013 PMC4116398

[phy214529-bib-0039] Mcilroy, G. D. , Tammireddy, S. R. , Maskrey, B. H. , Grant, L. , Doherty, M. K. , Watson, D. G. , … Mody, N. (2016). Fenretinide mediated retinoic acid receptor signalling and inhibition of ceramide biosynthesis regulates adipogenesis, lipid accumulation, mitochondrial function and nutrient stress signalling in adipocytes and adipose tissue. Biochemical Pharmacology, 100, 86–97. 10.1016/j.bcp.2015.11.017 26592777 PMC4762576

[phy214529-bib-0040] O’Neill, H. M. , Holloway, G. P. , & Steinberg, G. R. (2012). AMPK regulation of fatty acid metabolism and mitochondrial biogenesis: Implications for obesity. Molecular and Cellular Endocrinology, 366, 135–151. 22750049 10.1016/j.mce.2012.06.019

[phy214529-bib-0041] Parry, S. A. , Smith, J. R. , Corbett, T. R. B. , Woods, R. M. , & Hulston, C. J. (2017). Short‐term, high‐fat overfeeding impairs glycaemic control but does not alter gut hormone responses to a mixed meal tolerance test in healthy, normal‐weight individuals. British Journal of Nutrition, 117, 48–55. 10.1017/S0007114516004475 28115026

[phy214529-bib-0042] Parry, S. A. , Turner, M. C. , Woods, R. M. , James, L. J. , Ferguson, R. A. , Cocks, M. , … Hulston, C. J. . (2019). High‐fat overfeeding impairs peripheral glucose metabolism and muscle microvascular eNOS Ser1177 phosphorylation. The Journal of Clinical Endocrinology & Metabolism, 105(1), 65–77. 10.1210/clinem/dgz018. 31513265

[phy214529-bib-0043] Perry, C. G. R. , Talanian, J. L. , Heigenhauser, G. J. F. , & Spriet, L. L. (2007). The effects of training in hyperoxia vs. normoxia on skeletal muscle enzyme activities and exercise performance. Journal of Applied Physiology, 102, 1022–1027. 17170202 10.1152/japplphysiol.01215.2006

[phy214529-bib-0044] Rena, G. , Hardie, D. G. , & Pearson, E. R. (2017). The mechanisms of action of metformin. Diabetologia, 60, 1577–1585. 10.1007/s00125-017-4342-z 28776086 PMC5552828

[phy214529-bib-0045] Salminen, A. , Hyttinen, J. M. T. , & Kaarniranta, K. (2011). AMP‐activated protein kinase inhibits NF‐κB signaling and inflammation: Impact on healthspan and lifespan. Journal of Molecular Medicine, 89, 667–676. 10.1007/s00109-011-0748-0 21431325 PMC3111671

[phy214529-bib-0046] Samocha‐Bonet, D. , Campbell, L. V. , Mori, T. A. , Croft, K. D. , Greenfield, J. R. , Turner, N. , & Heilbronn, L. K. (2012). Overfeeding reduces insulin sensitivity and increases oxidative stress, without altering markers of mitochondrial content and function in humans. PLoS One, 7, e36320 10.1371/journal.pone.0036320 22586466 PMC3346759

[phy214529-bib-0047] Schubert, K. M. , Scheid, M. P. , & Duronio, V. (2000). Ceramide inhibits protein kinase B/Akt by promoting dephosphorylation of serine 473. Journal of Biological Chemistry, 275, 13330–13335. 10788440 10.1074/jbc.275.18.13330

[phy214529-bib-0048] Smith, G. I. , Atherton, P. , Reeds, D. N. , & Mohammed, B. S. , Rankin, D. , Rennie, M. J. , & Mittendorfer, B. (2011). Dietary omega‐3 fatty acid supplementation increases the rate of muscle protein synthesis in older adults : A randomized controlled trial. 1–3. 402–412. 10.3945/ajcn.110.005611 PMC3021432 21159787

[phy214529-bib-0049] Souza, D. R. D. , Pieri, B. L. D. S. , Comim, V. H. , Marques, S. D. O. , Luciano, T. F. , Rodrigues, M. S. , & De Souza, C. T. (2020). Fish oil reduces subclinical inflammation, insulin resistance, and atherogenic factors in overweight/obese type 2 diabetes mellitus patients: A pre‐post pilot study. Journal of Diabetes and Its Complications, 34(5), 107553 10.1016/j.jdiacomp.2020.107553 32014347

[phy214529-bib-0050] Steinberg, G. R. , Michell, B. J. , van Denderen, B. J. W. , Watt, M. J. , Carey, A. L. , Fam, B. C. , … Kemp, B. E. (2006). Tumor necrosis factor alpha‐induced skeletal muscle insulin resistance involves suppression of AMP‐kinase signaling. Cell Metabolism, 4, 465–474. 17141630 10.1016/j.cmet.2006.11.005

[phy214529-bib-0051] Straczkowski, M. , Kowalska, I. , Nikolajuk, A. , Dzienis‐Straczkowska, S. , Kinalska, I. , Baranowski, M. , … Gorski, J. (2004). Relationship between insulin sensitivity and sphingomyelin signaling pathway in human skeletal muscle. Diabetes, 53, 1215–1221. 10.2337/diabetes.53.5.1215 15111489

[phy214529-bib-0052] Talanian, J. L. , Galloway, S. D. R. , Heigenhauser, G. J. F. , Bonen, A. , & Spriet, L. L. (2007). Two weeks of high‐intensity aerobic interval training increases the capacity for fat oxidation during exercise in women. Journal of Applied Physiology, 102, 1439–1447. 10.1152/japplphysiol.01098.2006 17170203

[phy214529-bib-0053] Toledo, F. G. S. , Johannsen, D. L. , Covington, J. D. , Bajpeyi, S. , Goodpaster, B. , Conley, K. E. , & Ravussin, E. (2018). Impact of prolonged overfeeding on skeletal muscle mitochondria in healthy individuals. Diabetologia, 61, 466–475. 10.1007/s00125-017-4496-8 29150696 PMC5770194

[phy214529-bib-0054] Turner, N. , Bruce, C. R. , Beale, S. M. , Hoehn, K. L. , So, T. , Rolph, M. S. , & Cooney, G. J. (2007). Excess lipid availability increases mitochondrial fatty acid oxidative capacity in muscle. Diabetes, 56, 2085–2092. 10.2337/db07-0093 17519422

[phy214529-bib-0055] Turvey, E. A. , Heigenhauser, G. J. F. , Parolin, M. , & Peters, S. J. (2005). Elevated n‐3 fatty acids in a high‐fat diet attenuate the increase in PDH kinase activity but not PDH activity in human skeletal muscle. Journal of Applied Physiology, 98, 350–355. 10.1152/japplphysiol.00604.2004 15591305

[phy214529-bib-0056] Van Dam, R. M. , Rimm, E. B. , Willett, W. C. , Stampfer, M. J. , & Hu, F. B. (2002). Dietary patterns and risk for type 2 diabetes mellitus in U.S. men. Annals of Internal Medicine, 136, 201–209. 10.7326/0003-4819-136-3-200202050-00008 11827496

[phy214529-bib-0057] Zabielski, P. , Daniluk, J. , Hady, H. R. , Markowski, A. R. , Imierska, M. , Górski, J. , & Blachnio‐Zabielska, A. U. (2019). The effect of high‐fat diet and inhibition of ceramide production on insulin action in liver. Journal of Cellular Physiology, 234, 1851–1861. 10.1002/jcp.27058 30067865

[phy214529-bib-0058] Zierath, J. R. (2007). The path to insulin resistance: Paved with ceramides? Cell Metabolism, 5, 161–163. 10.1016/j.cmet.2007.02.005 17339023

