# Supplementary material for: Human skeletal muscle metabolic responses to 6 days of high‐fat overfeeding are associated with dietary n‐3PUFA content and muscle oxidative capacity
Source: Physiol Rep. 2020 Aug 26;8(16):e14529. doi: 10.14814/phy2.14529 (PMC7448800; doi:10.14814/phy2.14529)
Supplement: Supplementary file 1 — Table S1‐S2 [file PHY2-8-e14529-s001.docx]

SUPPLEMENTARY TABLES

| **Table 1: Blood lipid profiling by gas liquid chromatography** | | | | |
| --- | --- | --- | --- | --- |
|  | **HF-C** | | **HF-FO** | |
|  | **Pre-HFEE** | **Post-HFEE** | **Pre-HFEE** | **Post-HFEE** |
| 14:00 | 0.57 ± 0.13 | 0.66 ± 0.11 | 0.57 ± 0.13 | 0.67 ± 0.10 |
| 15:00 | 0.20 ± 0.03 | 0.24 ± 0.03* | 0.18 ± 0.03 | 0.24 ± 0.03* |
| 16:00 | 20.97 ± 0.69 | 20.69 ± 0.43 | 20.80 ± 0.75 | 20.44 ± 0.84 |
| 18:00 | 11.42 ± 0.23 | 11.51 ± 0.33 | 11.44 ± 0.71 | 12.03 ± 0.57* |
| 20:00 | 0.25 ± 0.02 | 0.26 ± 0.02 | 0.24 ± 0.02 | 0.25 ± 0.02 |
| 22:00 | 0.76 ± 0.10 | 0.80 ± 0.09 | 0.72 ± 0.08 | 0.76 ± 0.08 |
| 24:00 | 1.13 ± 0.07 | 1.24 ± 0.44 | 1.13 ± 0.11 | 1.12 ± 0.11 |
| **Total SFA** | **35.30 ± 0.91** | **35.38 ± 0.94** | **35.09 ± 1.34** | **35.51 ± 1.02** |
| 16:1n-9 | 0.27 ± 0.05 | 0.24 ± 0.04 | 0.26 ± 0.06 | 0.21 ± 0.05* |
| 16:1n-7 | 1.04 ± 0.26 | 0.77 ± 0.15* | 1.15 ± 0.35 | 0.80 ± 0.23* |
| 18:1n-9 | 17.29 ± 1.35 | 15.15 ± 1.52* | 16.61 ± 1.45 | 13.44 ± 0.91* |
| 18:1n-7 | 1.64 ± 0.13 | 1.44 ± 0.12* | 1.57 ± 0.26 | 1.42 ± 0.12 |
| 20:1n-9 | 0.25 ± 0.03 | 0.23 ± 0.03* | 0.24 ± 0.03 | 0.30 ± 0.05* |
| 24:1n-9 | 1.46 ± 0.14 | 1.43 ± 0.11 | 1.33 ± 0.16 | 1.43 ± 0.16* |
| **Total MUFA** | **21.95 ± 1.45** | **19.25 ± 1.74*** | **21.16 ± 1.92** | **17.61 ± 1.16*** |
| 18:2n-6 | 18.78 ± 2.58 | 21.97 ± 1.86* | 19.91 ± 2.20 | 18.30 ± 1.01* |
| 18:3n-6 | 0.22 ± 0.05 | 0.21 ± 0.08 | 0.22 ± 0.11 | 0.13 ± 0.04* |
| 20:2n-6 | 0.22 ± 0.02 | 0.22 ± 0.02 | 0.26 ± 0.04 | 0.25 ± 0.03 |
| 20:3n-6 | 1.73 ± 0.37 | 1.50 ± 0.26* | 1.71 ± 0.46 | 1.28 ± 0.28* |
| 20:4n-6 | 10.96 ± 1.07 | 10.73 ± 1.05 | 10.93 ± 1.15 | 10.77 ± 0.93 |
| 22:4n-6 | 1.51 ± 0.16 | 1.46 ± 0.13 | 1.60 ± 0.22 | 1.51 ± 0.24* |
| 22:5n-6 | 0.32 ± 0.05 | 0.30 ± 0.05* | 0.35 ± 0.08 | 0.32 ± 0.07* |
| **Total n-6 PUFA** | **33.74 ± 1.75** | **36.39 ± 1.15*** | **34.97 ± 1.84** | **32.54 ± 1.07*** |
| 18:3n-3 | 0.51 ± 0.09 | 0.53 ± 0.09 | 0.51 ± 0.14 | 0.55 ± 0.07 |
| 20:4n-3 | 0.08 ± 0.02 | 0.07 ± 0.01 | 0.12 ± 0.06 | 0.16 ± 0.06 |
| 20:5n-3 | 0.69 ± 0.18 | 0.73 ± 0.17 | 0.57 ± 0.14 | 3.95 ± 1.20* |
| 22:5n-3 | 1.57 ± 0.20 | 1.50 ± 0.19* | 1.40 ± 0.20 | 1.56 ± 0.14* |
| 22:6n-3 | 2.58 ± 0.56 | 2.55 ± 0.49 | 2.51 ± 0.44 | 4.21 ± 0.58* |
| **Total n-3 PUFA** | **5.41 ± 0.83** | **5.35 ± 0.74** | **5.04 ± 0.51** | **10.43 ± 1.61*** |
| 16:0DMA | 1.26 ± 0.10 | 1.30 ± 0.14 | 1.33 ± 0.10 | 1.42 ± 0.07* |
| 18:0DMA | 1.76 ± 0.11 | 1.75 ± 0.17 | 1.83 ± 0.20 | 1.88 ± 0.21 |
| 18:1DMA | 0.58 ± 0.06 | 0.58 ± 0.05 | 0.57 ± 0.07 | 0.61 ± 0.08* |
| **Total DMA** | **3.60 ± 0.22** | **3.63 ± 0.31** | **3.74 ± 0.25** | **3.92 ± 0.26*** |
| **20:4n-6/20:5n-3** | **16.66 ± 3.71** | **15.23 ± 2.83** | **20.14 ± 4.98** | **3.01 ± 1.06*** |
| **% n-3HUFA/Total HUFA** | **24.87 ± 2.75** | **25.35 ± 2.36** | **23.46 ± 2.67** | **40.96 ± 4.72*** |
| All individual fatty acids are expressed as a percentage of total fatty acids. Values are means ± SD (n = 10 per group), where HFEE = high-fat energy excess; HF-C = high-fat control; HF-FO = high-fat fish oil. * indicates significantly different (P < 0.05) from Pre-HFEE within group. | | | | |

| **Table 2. Skeletal muscle lipid profiling by GCMS** | | | | |
| --- | --- | --- | --- | --- |
|  | **HF-C** | | **HF-FO** | |
|  | **Pre-HFEE** | **Post-HFEE** | **Pre-HFEE** | **Post-HFEE** |
| 14:0 | 0.78 ± 0.37 | 0.76 ± 0.32 | 0.82 ± 0.24 | 1.18 ± 0.48 |
| 15:0 | 0.67 ± 0.38 | 0.67 ± 0.39 | 0.56 ± 0.12 | 0.79 ± 0.26* |
| 16:0 | 20.41 ± 2.97 | 20.73 ± 2.59 | 20.91 ± 1.97 | 21.16 ± 2.30 |
| 18:0 | 13.18 ± 2.39 | 12.74 ± 2.50 | 13.32 ± 2.21 | 12.96 ± 2.07 |
| 20:0 | 0.14 ± 0.04 | 0.12 ± 0.04 | 0.16 ± 0.07 | 0.18 ± 0.12 |
| 22:0 | 0.23 ± 0.13 | 0.20 ± 0.07 | 0.24 ± 0.10 | 0.25 ± 0.12 |
| 24:0 | 0.20 ± 0.11 | 0.20 ± 0.10 | 0.22 ± 0.07 | 0.20 ± 0.11 |
| **Total SFA** | **35.61 ± 1.82** | **35.42 ± 0.94** | **36.23 ± 2.74** | **36.71 ± 2.68** |
| 16:1n-9 | 0.57 ± 0.82 | 0.29 ± 0.13 | 0.39 ± 0.21 | 0.40 ± 0.20 |
| 16:1n-7 | 0.59 ± 0.44 | 0.60 ± 0.36 | 0.81 ± 0.43 | 0.85 ± 0.75 |
| 18:1n-9 | 8.09 ± 1.10 | 7.70 ± 1.09 | 8.25 ± 1.27 | 8.20 ± 1.49 |
| 18:1n-7 | 1.83 ± 0.23 | 1.75 ± 0.15 | 1.80 ± 0.33 | 1.88 ± 0.36 |
| 20:1n-11 | 0.04 ± 0.06 | 0.04 ± 0.06 | 0.05 ± 0.08 | 0.10 ± 0.11 |
| 20:1n-9 | 0.24 ± 0.24 | 0.29 ± 0.31 | 0.42 ± 0.58 | 0.68 ± 0.59 |
| 22:1n-11 | 0.24 ± 0.37 | 0.30 ± 0.44 | 0.32 ± 0.46 | 0.60 ± 0.78 |
| 22:1n-9 | 0.12 ± 0.07 | 0.10 ± 0.08 | 0.16 ± 0.09 | 0.18 ± 0.11 |
| 24:1n-9 | 0.48 ± 0.29 | 0.43 ± 0.14 | 0.56 ± 0.21 | 0.66 ± 0.45 |
| **Total MUFA** | **12.21 ± 1.88** | **11.51 ± 2.15** | **12.76 ± 2.97** | **13.54 ± 4.00** |
| 18:2n-6 | 29.44 ± 2.45 | 30.72 ± 3.75 | 28.60 ± 3.12 | 26.69 ± 4.13 |
| 18:3n-6 | 0.17 ± 0.08 | 0.13 ± 0.03 | 0.18 ± 0.05 | 0.16 ± 0.05 |
| 20:2n-6 | 0.12 ± 0.03 | 0.12 ± 0.04 | 0.16 ± 0.08 | 0.18 ± 0.08 |
| 20:3n-6 | 1.53 ± 0.29 | 1.49 ± 0.29 | 1.46 ± 0.27 | 1.34 ± 0.38 |
| 20:4n-6 | 14.04 ± 2.50 | 13.69 ± 2.24 | 13.79 ± 1.90 | 12.67 ± 1.95 |
| 22:4n-6 | 0.74 ± 0.17 | 0.74 ± 0.10 | 0.69 ± 0.19 | 0.58 ± 0.15* |
| 22:5n-6 | 0.41 ± 0.10 | 0.35 ± 0.09 | 0.41 ± 0.16 | 0.34 ± 0.11 |
| **Total n-6 PUFA** | **46.44 ± 3.30** | **47.25 ± 3.86** | **45.29 ± 4.79** | **41.96 ± 6.06** |
| 18:3n-3 | 0.36 ± 0.08 | 0.37 ± 0.06 | 0.34 ± 0.07 | 0.39 ± 0.11 |
| 18:4n-3 | 0.08 ± 0.13 | 0.13 ± 0.20 | 0.10 ± 0.18 | 0.10 ± 0.30 |
| 20:4n-3 | 0.06 ± 0.05 | 0.06 ± 0.06 | 0.07 ± 0.06 | 0.11 ± 0.09 |
| 20:5n-3 | 1.05 ± 0.45 | 1.08 ± 0.51 | 1.01 ± 0.45 | 1.87 ± 0.95* |
| 22:5n-3 | 1.86 ± 0.28 | 1.87 ± 0.26 | 1.61 ± 0.65 | 1.91 ± 0.30 |
| 22:6n-3 | 2.32 ± 0.60 | 2.31 ± 0.76 | 2.59 ± 1.15 | 3.41 ± 1.78 |
| **Total n-3 PUFA** | **5.73 ± 1.42** | **5.83 ± 1.62** | **5.73 ± 1.52** | **7.79 ± 3.22** |
| **20:4n-6/20:5n-3** | 16.22 ± 7.96 | 15.71 ± 8.07 | 16.83 ± 9.60 | 8.37 ± 4.02* |
| **% n-3HUFA/Total HUFA** | 24.05 ± 6.30 | 24.44 ± 5.65 | 24.29 ± 5.94 | 32.04 ± 10.08* |
|  |  |  |  |  |
| All individual fatty acids are expressed as a percentage of total fatty acids. Values are means ± SD (n = 9 per group), where HFEE = high-fat energy excess; HF-C = high-fat control; HF-FO = high-fat fish oil. * indicates significantly different (P<0.05) from Pre-HFEE within group. | | | | |
